# A Novel Membrane Sensor Controls the Localization and ArfGEF Activity of Bacterial RalF

**DOI:** 10.1371/journal.ppat.1003747

**Published:** 2013-11-14

**Authors:** Marcia Folly-Klan, Eric Alix, Danièle Stalder, Pampa Ray, Lionel V. Duarte, Anna Delprato, Mahel Zeghouf, Bruno Antonny, Valérie Campanacci, Craig R. Roy, Jacqueline Cherfils

**Affiliations:** 1 Laboratoire d'Enzymologie et Biochimie Structurales, Centre de Recherche de Gif, CNRS, Gif-sur-Yvette, France; 2 Department of Microbial Pathogenesis, Yale University School of Medicine, New Haven, Connecticut, United States of America; 3 Institut de Pharmacologie Moléculaire et Cellulaire, Université de Nice-Sophia Antipolis et CNRS, Valbonne, France; University of Michigan, United States of America

## Abstract

The intracellular bacterial pathogen *Legionella pneumophila* (Lp) evades destruction in macrophages by camouflaging in a specialized organelle, the *Legionella*-containing vacuole (LCV), where it replicates. The LCV maturates by incorporating ER vesicles, which are diverted by effectors that Lp injects to take control of host cell membrane transport processes. One of these effectors, RalF, recruits the trafficking small GTPase Arf1 to the LCV. LpRalF has a Sec7 domain related to host ArfGEFs, followed by a capping domain that intimately associates with the Sec7 domain to inhibit GEF activity. How RalF is activated to function as a LCV-specific ArfGEF is unknown. We combined the reconstitution of Arf activation on artificial membranes with cellular expression and Lp infection assays, to analyze how auto-inhibition is relieved for LpRalF to function *in vivo*. We find that membranes activate LpRalF by about 1000 fold, and identify the membrane-binding region as the region that inhibits the Sec7 active site. It is enriched in aromatic and positively charged residues, which establish a membrane sensor to control the GEF activity in accordance with specific lipid environments. A similar mechanism of activation is found in RalF from *Rickettsia prowazekii* (Rp), with a different aromatic/charged residues ratio that results in divergent membrane preferences. The membrane sensor is the primary determinant of the localization of LpRalF on the LCV, and drives the timing of Arf activation during infection. Finally, we identify a conserved motif in the capping domain, remote from the membrane sensor, which is critical for RalF activity presumably by organizing its active conformation. These data demonstrate that RalF proteins are regulated by a membrane sensor that functions as a binary switch to derepress ArfGEF activity when RalF encounters a favorable lipid environment, thus establishing a regulatory paradigm to ensure that Arf GTPases are efficiently activated at specific membrane locations.

## Author Summary

The intracellular pathogens *Legionella pneumophila* (Lp) and *Rickettsia prowazekii* (Rp) inject an effector (RalF) that diverts the host trafficking small GTPase Arf1. In the case of Lp, LpRalF recruits Arf1 to the *Legionella*-containing vacuole (LCV), where the pathogen replicates. RalF proteins are related to eukaryotic ArfGEFs, from which they depart by a unique auto-inhibitory capping domain that contains localization and functional determinants. In this work, we combined the reconstitution of RalF ArfGEF activity on artificial membranes with cellular and Lp infection assays to uncover how auto-inhibition is released for RalF proteins to function in vivo. We find that LpRalF and RpRalF are activated by membranes by about 1000-fold and map the membrane sensor to a unique motif in the capping domain. This motif is identical to the auto-inhibitory motif, thus establishing a binary switch that controls the ArfGEF activity of RalF in accordance with specific lipid environments. Finally, we show that the membrane sensor drives LpRalF binding to the LCV and timing of Arf activation during Lp infection. These results establish how RalF proteins are derepressed when they encounter a favorable lipid environment, and provide an evolutionary explanation to the presence of RalF in pathogens with diverging lifestyles.

## Introduction

A number of intracellular pathogenic bacteria can bypass regulatory networks used to control trafficking and cytoskeletal pathways of the infected cell by delivering bacterial effector proteins into the host cytosol that function as illegitimate regulators of small GTPases (reviewed in [1], [2]). One of them, *Legionella pneumophila* (Lp), the causative agent of a severe pneumonia, the Legionnaire's disease, invades and replicates in macrophages where it survives in a specialized membrane-bound compartment, the *Legione*lla-containing vacuole (LCV) (reviewed in [3]). Maturation of the phagosome into the LCV is driven by an arsenal of effectors delivered by a type IV secretion system called Dot/Icm [4], [5]. Instead of fusing with lysosomes where degradative enzymes would destroy the pathogen, the LCV incorporates membranes from the endoplasmic reticulum (ER), a nutrient-rich compartment that supports multiplication of Lp in high numbers within the macrophage [3], [6], [7]. Over the last decade, a number of Lp effectors have been shown to divert cellular proteins that steer membrane traffic (reviewed in [2], [3], [8]. These include several illegitimate regulators or modifiers of small GTPases of the Arf and Rab families, which are major regulators of cellular traffic in eukaryotes (reviewed in [9], [10]).

One of these effector proteins, RalF, contains a Sec7 homology region [4], which is the catalytic domain in eukaryotic guanine nucleotide exchange factors (GEFs) that is sufficient to activate Arf by stimulating GDP/GTP exchange (reviewed in [11]). Shortly after infection, the LpRalF protein is detected on the cytosolic surface of limiting membranes that defines the LCV [4]. Localization of LpRalF to the LCV is sufficient to mediate the recruitment of cellular Arf GTPases to this organelle by a mechanism that is dependent on a functional Sec7 domain. Arf activity is important for fusion of ER-derived membranes with the LCV [7], although the recruitment of Arf proteins to the LCV is currently of unknown importance (reviewed in [3]). A protein with primary sequence similarity to LpRalF is also encoded by *Rickettsia prowazekii* (Rp) [4], the bacterial pathogen responsible for epidemic typhus. Rp is unrelated to Lp phylogenetically, and unlike Lp, it lyses the vacuole in which it resides to replicate freely in the cytosol (reviewed in [12]).

Structural studies showed that the C-terminal domain of LpRalF intimately associates with the Sec7 domain to block access to the Arf-binding site [13]. Accordingly, the ArfGEF activities of LpRalF and its homolog from *Rickettsia* are strongly auto-inhibited *in vitro* [4], [13], [14]. This domain was thus termed the capping domain. Recently, the capping domains of LpRalF and RpRalF were shown to localize to host membranes when expressed in cells [14]. However, the LpRalF capping domain localized to a perinuclear region reminiscent of the endoplasmic reticulum, whereas the RpRalF capping domain localized to the plasma membrane. In addition, expression of full-length LpRalF and RpRalF in cells resulted in divergent effects, with LpRalF impairing secretion and RpRalF disrupting actin dynamics at the plasma membrane [14]. Thus, the LpRalF and RpRalF proteins represent similar ArfGEF proteins that display different functions and membrane localization properties.

The mechanism by which the LpRalF protein identifies the LCV membrane and restricts its ArfGEF activity to this organelle inside the host cell, and how LpRalF and RpRalf target different membranes, remain important and unanswered questions. In eukaryotes, evidence has accumulated that GEFs not only activate their small GTPase substrates by stimulating GDP/GTP exchange, but also process upstream activating signals, restrict the subcellular localization of active GTPases, and likely convey downstream information (reviewed in [15]). These multiple functions are controlled by sophisticated regulatory mechanisms such as auto-inhibition, feed-back loops and activating interactions with other proteins and/or with membranes, and often involve large conformational changes. Although the auto-inhibitory capping domain of RalF proteins is unrelated to any domain of known structure or function, localization studies in cells have suggested that it has a critical role in RalF localization, and that membrane interactions may be needed to relieve autoinhibition and regulate the ArfGEF activity of the LpRalF protein in vivo.

Here, we investigated the RalF nucleotide exchange reaction using artificial membranes to reconstitute the cellular environment in which the LpRalF and RpRalF proteins function. Combined with cellular expression and Lp infection assays, these data have led to the identification of a membrane sensor in the capping domain of RalF proteins that contributes to membrane localization and spatial regulation of Arf activation on membranes during infection of host cells.

## Results

### Membrane-driven activation of Legionella RalF

**Figure 1:**
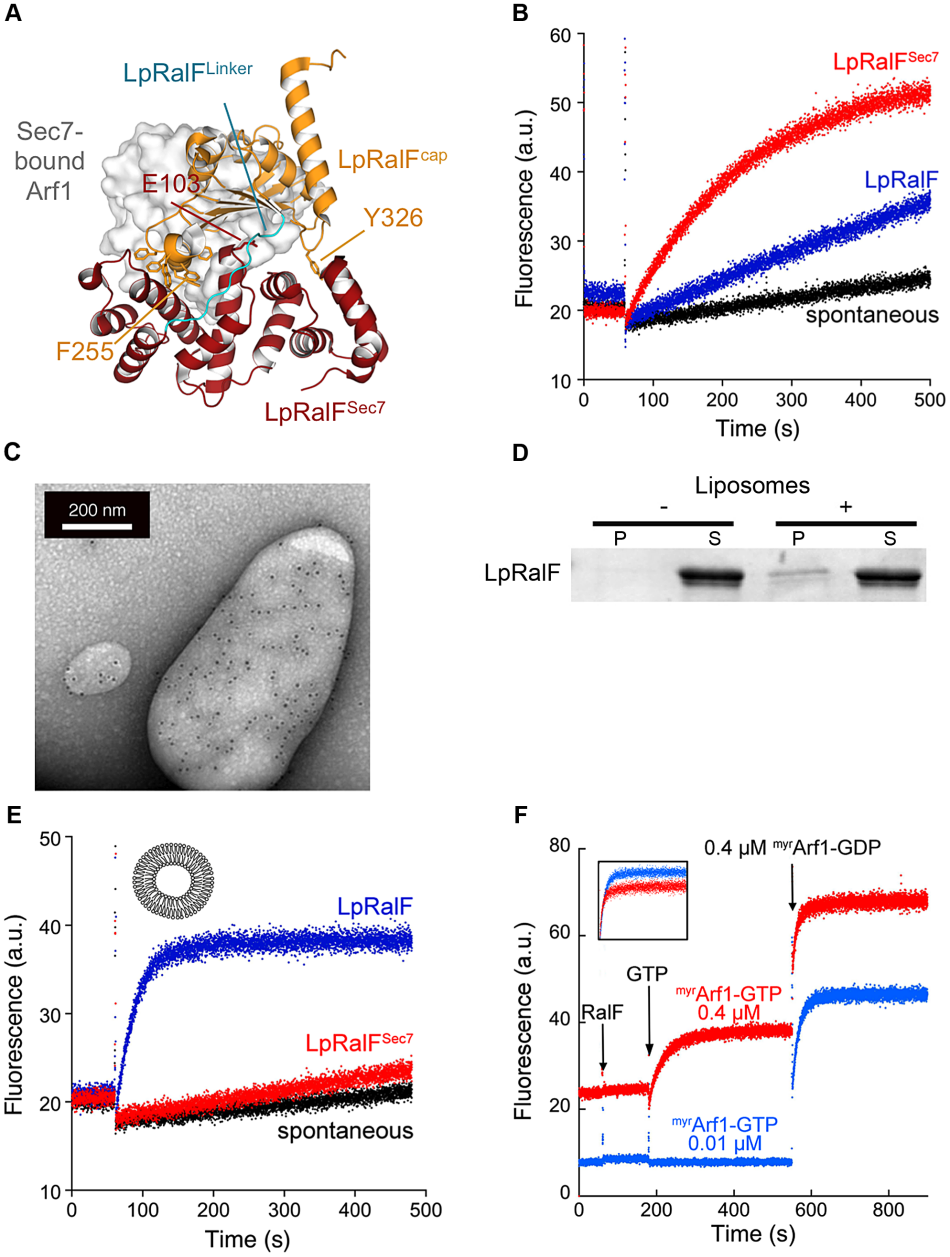
*Legionella* RalF is activated by membranes. A. Structure of auto-inhibited *Legionella pneumophila* RalF. The Sec7 domain (in red) and capping domain (in orange) are connected by a 10-residue linker (in cyan). The structure of nucleotide-free Arf1 bound to a yeast Sec7 domain is overlaid (in surface representation; from [22]), highlighting the structural blockage of the GEF active site by the capping domain. Drawn from PDB entry 1XSZ [13]. **B.** **Representative nucleotide exchange kinetics of Arf1 activation by LpRalF in solution.** Nucleotide exchange was monitored by tryptophan fluorescence (a.u. arbitrary units) for Δ17Arf1 (1 µM) alone and in the presence of RalF constructs (1 µM) as indicated. All experiments were started by addition of 100 µM GTP. **C.** **Immunogold labeling electron microscopy of LpRalF bound to liposomes.** His-tagged LpRalF labeled with anti-His antibody in the presence of extruded liposomes was detected with a 10 nm gold anti-mouse antibody (black dots). **D.** **Co-sedimentation of LpRalF with liposomes** containing 39% PC, 20% PE, 25% PS, 1% PIP_2_, 15% cholesterol. P = pellet, S = supernatant. **E.** **Representative nucleotide exchange kinetics of ^myr^Arf1 activation by LpRalF in the presence of liposomes.** ^myr^Arf1 (0.4 µM) and the indicated LpRalF constructs (0.1 µM) were assayed in the presence of 200 µM liposomes (composition as in **Figure 1D**). **F.** **LpRalF is not regulated by a feed-back loop.** Increasing amounts of ^myr^Arf1-GTP were pre-formed in the presence of LpRalF (0.1 µM) until the plateau was reached. Then, the exchange rate was measured after a second addition of ^myr^Arf1-GDP (0.4 µM). The inset shows the overlay of the second part of the reaction after correction for intrinsic fluorescence, from which k_obs_ were calculated.

The C-terminal capping domain of LpRalF obstructs the active site of its Sec7 domain, hence must be displaced to bind Arf GTPase substrates (**Figure 1A**). We first used purified full-length LpRalF and its Sec7 domain alone (LpRalF^Sec7^, residues 1–201) (**Figure S1A**) to quantify the auto-inhibition of nucleotide exchange by the capping domain. Nucleotide exchange kinetics of human Arf1 lacking its N-terminal α-helix (Δ17Arf1), which is readily activated by ArfGEFs in solution (reviewed in [16]), were monitored by tryptophan fluorescence (**Figure 1B**). As previously reported [13], [14], LpRalF was essentially inactive in solution (k_cat_/K_m_ = 3.15±0.17 10^2^ M^−1^s^−1^), reflecting strong auto-inhibition. Removal of its capping domain increased exchange efficiency by about 10-fold (k_cat_/K_m_ = 4.41±0.52 10^3^ M^−1^s^−1^). Detection of measurable nucleotide exchange required at least stoechiometric LpRalF amounts, and remained about 1–2 orders of magnitude lower than what is achieved by cellular ArfGEFs [17]. LpRalF^Sec7^ has however the hallmarks of a conventional Sec7 domain, as it readily formed exchange intermediates with Arf1-GDP by mutating the catalytic glutamate to lysine (E103K) (**Figure S1B**) and with nucleotide-free Arf1 by enzymatic removal of GDP (**Figure S1C**).

These data suggest that other factors are needed for LpRalF to reach full efficiency on the LCV. The capping domain of LpRalF was previously shown to co-localize with subcellular compartments and to fractionate with membranes when expressed in cells [14], suggesting that membranes could be involved in activation. We first analyzed whether full-length LpRalF has the ability to bind to membranes. Direct binding to liposomes was observed using immunogold-labeling electron microscopy (**Figure 1C**) and by liposome co-sedimentation experiments (**Figure 1D**). Next, we conducted ArfGEF assays *in vitro* in the presence of synthetic liposomes using purified LpRalF and the cellular form of Arf1, which carries a myristoyl lipid attached to its N-terminal helix (^myr^Arf1) (**Figure 1E**). Using this more physiological ArfGEF assay having both ^myr^Arf1 and liposomes the catalytic efficiency of LpRalF increased by 3 orders of magnitude compared to its activity in solution (k_cat_/K_m_ = 2.44±0.10 10^5^ M^−1^s^−1^), thus reaching an efficiency similar to that of eukaryotic ArfGEFs [17]. Importantly, under these physiological conditions, LpRalF^Sec7^ remained essentially inactive (**Figure 1E**), which indicates that the Sec7 domain alone is unable to efficiently activate ^myr^Arf1 on a membrane surface and that the potentiating effect is entirely mediated by the capping domain.

Recent studies highlighted positive feedback regulation of cellular GEFs on membranes, in which initial production of the GTP-bound GTPase potentiates nucleotide exchange efficiency [18]–[20]. To analyze whether LpRalF is controlled by a feedback loop, we either added a large amount of the Arf effector GRAB to the exchange reaction to cancel a feedback effect by depleting Arf1-GTP as it is generated, or we added increasing amounts of ^myr^Arf1-GTP prior to measuring exchange rates to maximize such an effect. Neither of these conditions affected the exchange rate of LpRalF (**Figures S1D and 1F**), indicating that LpRalF is not under a positive feedback control by ^myr^Arf1-GTP.

Together, these results suggest that RalF activation comprises a conformational component, which is required to release its auto-inhibition by the capping domain, and a spatial component that is needed for its localization to membranes. These components add up to yield an activation of about 1000-fold, of which the conformational change of the capping domain accounts for about a 10-fold contribution as mimicked in solution by the deletion of the capping domain. Our data also show that neither the Sec7 domain nor membrane-attached ^myr^Arf1-GTP contribute to the spatial component, indicating that potentiation of LpRalF activity by membranes is entirely mediated by its capping domain.

### The aromatic cluster in the capping domain is a novel membrane sensor

**Figure 2:**
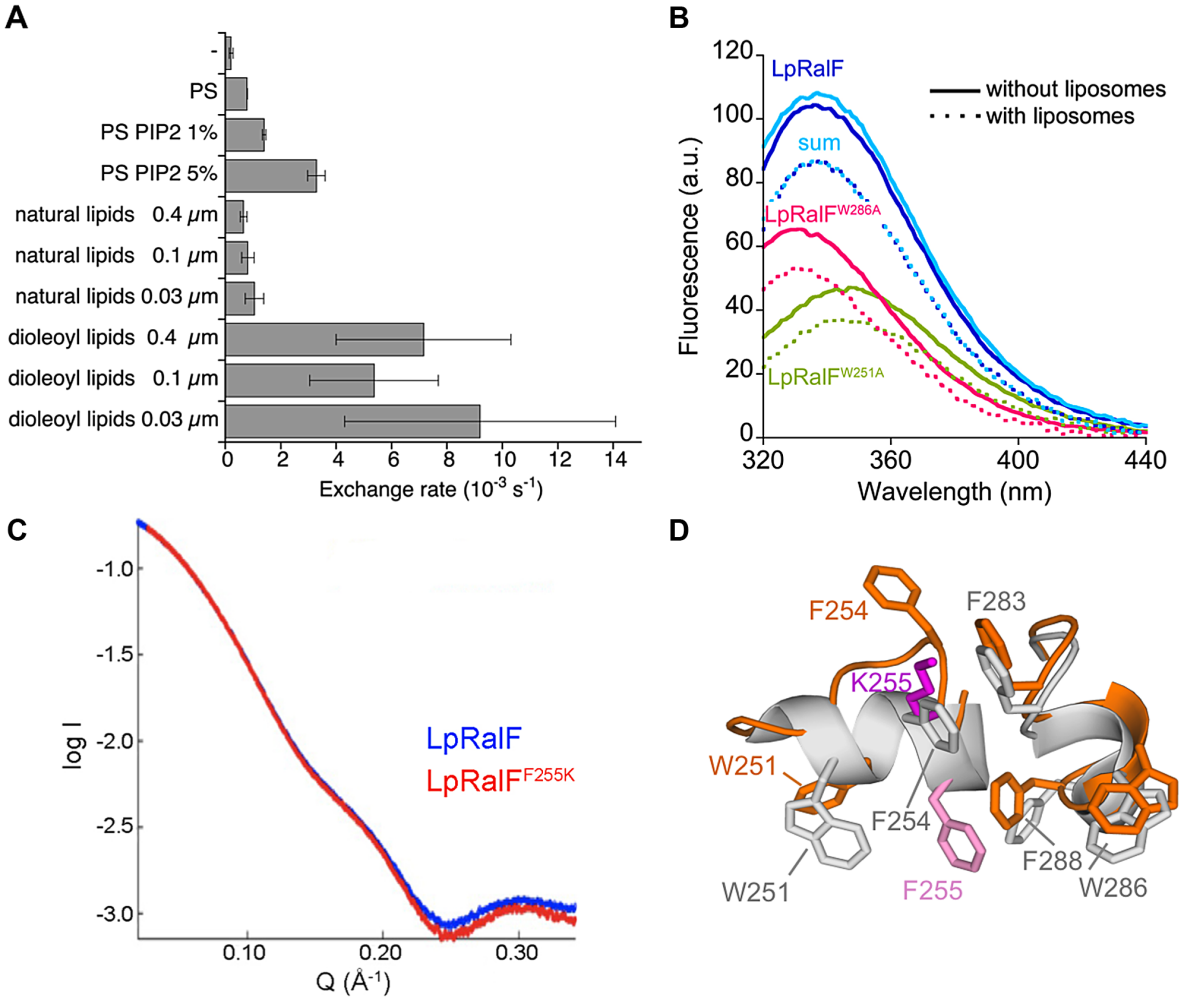
The aromatic cluster in the capping domain of LpRalF is a membrane sensor. A. Nucleotide exchanged rates analyzed in the presence of liposomes of different composition, curvature and packing as indicated. All nucleotide exchange experiments were carried out with ^myr^Arf1 (0.4 µM) and LpRalF (0.1 µM) in the presence of 200 µM liposomes and monitored by Trp fluorescence. Representative fluorescence recordings are shown in **Figure S2A and S2B**. **B.** **Interaction of LpRalF with liposomes analyzed by tryptophan fluorescence.** Fluorescence scans done without and with liposomes are in plain and dotted lines, respectively. The sum of the tryptophan fluorescence scans of the mutants is shown in light blue. Composition of liposomes is as in **Figure 1D**. **C.** **Synchrotron SAXS profiles** of wild-type LpRalF (blue) and LpRalF^F255K^ (red). **D.** **Crystal structure of LpRalF^F255K^.** Close-up view of the aromatic cluster of LpRalF^F255K^ (in orange, K255 in magenta) superimposed to wild-type LpRalF (in grey, F255 in light magenta). The structure of LpRalF^F255K^ is shown in **Figure S2C**.

The recognition of the capping domain of LpRalF as a membrane-binding domain without homology to any known membrane-binding determinants prompted us to investigate its sensitivity to membranes physico-chemical properties and curvature. This was probed by comparing the exchange rates at fixed concentrations of LpRalF (0.1 µM) and ^myr^Arf1 (0.4 µM), using the fluorescence-based nucleotide exchange assay in the presence of liposomes of various charges, curvature and packing (**Figure 2A** **, S2A and S2B**). LpRalF had only a weak activity in the presence of uncharged liposomes containing only phosphatidylcholine (PC) and phosphatidylethanolamine (PE). Exchange rates were increased up to 15-fold in the presence of negatively charged phospholipids, with the largest effects resulting from addition of 30% phosphatidylserine (PS) and 5% phosphatidylinositol (4,5) bisphosphate (PIP_2_). Liposomes of intermediate charge characteristics (50% PC, 19% PE, 5% PS, 10% phosphatidylinositol (PI), 16% cholesterol) extruded through filters of different sizes were used to probe the effect of curvature and packing. Increasing the curvature using filters from 0.4 µm to 0.03 µm resulted only in a modest increase of the exchange rates. In contrast, replacing natural PC, PE and PS lipids by dioleoyl-PC, dioleoyl-PE and dioleoyl-PS lipids, whose unsaturated fatty acid chains form more loosely packed membranes, resulted in a 10-fold increase of the exchange rates. These results indicate that LpRalF is highly sensitive to both the presence of negatively charged lipids and to packing defects but is not sensitive to membrane curvature.

A prominent feature of LpRalF^cap^ is an unusual cluster of aromatic residues, located on two twin α-helices, ^248^PKSWLSFFTG^257^ and ^280^PNIFSKWLFG^289^, which are wedged into the catalytic groove of the Sec7 domain and must be displaced before Arf GTPases can interact with the Sec7 catalytic site (**Figure 1A**). Aromatic residues are well-suited for peripheral insertion of proteins into membranes [21], suggesting that the membrane-binding site of the capping domain may encompass these helices. We used Trp fluorescence to measure whether addition of liposomes result in changes in the environment of Trp251 and Trp286, which are located in the cluster. Liposomes induced a marked decrease in the fluorescence signal, to which Trp251 and Trp286 contributed in an additive manner as shown by their mutations to alanines (**Figure 2B**). These data suggest that the environment of the aromatic cluster is modified by the presence of membranes. However, they do not discriminate between effects that are due to auto-inhibition release from those arising from a potential interaction with membranes. Alternatively, we reasoned that introducing a charged residue in the cluster could reveal direct membrane interactions by monitoring nucleotide exchange in the presence of negatively charged lipids. We chose to mutate Phe255, which has a strategic location in auto-inhibited LpRalF, where it mimics a critical Phe residue in the switch 1 of Arf (Phe51) [22], [23] and is superimposable to an auto-inhibitory Phe residue in cytohesins (Phe 262 in the PH domain of GRP1, [24]). We first assessed whether the F255K mutation would affect the biochemical and structural properties of LpRalF in solution. The SAXS profiles of LpRalF and LpRalF^F255K^ were in good agreement at small Q values (<0.2 Å^−1^), indicating that LpRalF^F255K^ is mostly in an auto-inhibited conformation in solution (**Figure 2C**). Consistently, the crystal structure of LpRalF^F255K^ retained an auto-inhibited conformation (**Figure S2C**), in which the mutation is accommodated by increased local disorder (**Figure 2D**). In agreement with structural data, LpRalF^F255K^ remained auto-inhibited in solution (k_obs_ = 2.5±0.9 10^−4^ s^−1^ measured at 1 µM LpRalF^F255K^ and 1 µM Δ17Arf1, to be compared with k_obs_ = 9±0.9 10^−4^ s^−1^ measured for LpRalF under the same conditions). These data establish that the F255K mutation is silent in solution, thus that this protein can be used to investigate auto-inhibition and membrane effects independently. Consistent with the hypothesis, LpRalF^F255K^ was at least 10 times more active than wild type LpRalF in the presence of negatively charged liposomes (k_obs_ = 47.3±12 10^−2^ s^−1^ measured at 0.1 µM LpRalF^F255K^ and 0.4 µM ^myr^Arf1) (**Figure S2D**), suggesting that the extra lysine residue facilitates interactions with negatively-charged lipids. Conversely, we reasoned that mutation of F255 to glutamate should introduce repulsive interactions with negatively-charged lipids that should be detrimental to its efficiency. Indeed the nucleotide exchange activity of LpRalF^F255E^ was reduced 4-fold compared to wild-type LpRalF (k_obs_ = 1.3±0.2 10^−2^ s^−1^) (**Figure S2D**). These large effects observed in the presence of membranes but not in solution strongly suggest that the membrane-binding site of the capping domain encompasses the aromatic cluster.

### Rickettsia RalF is a membrane-activated ArfGEF

**Figure 3:**
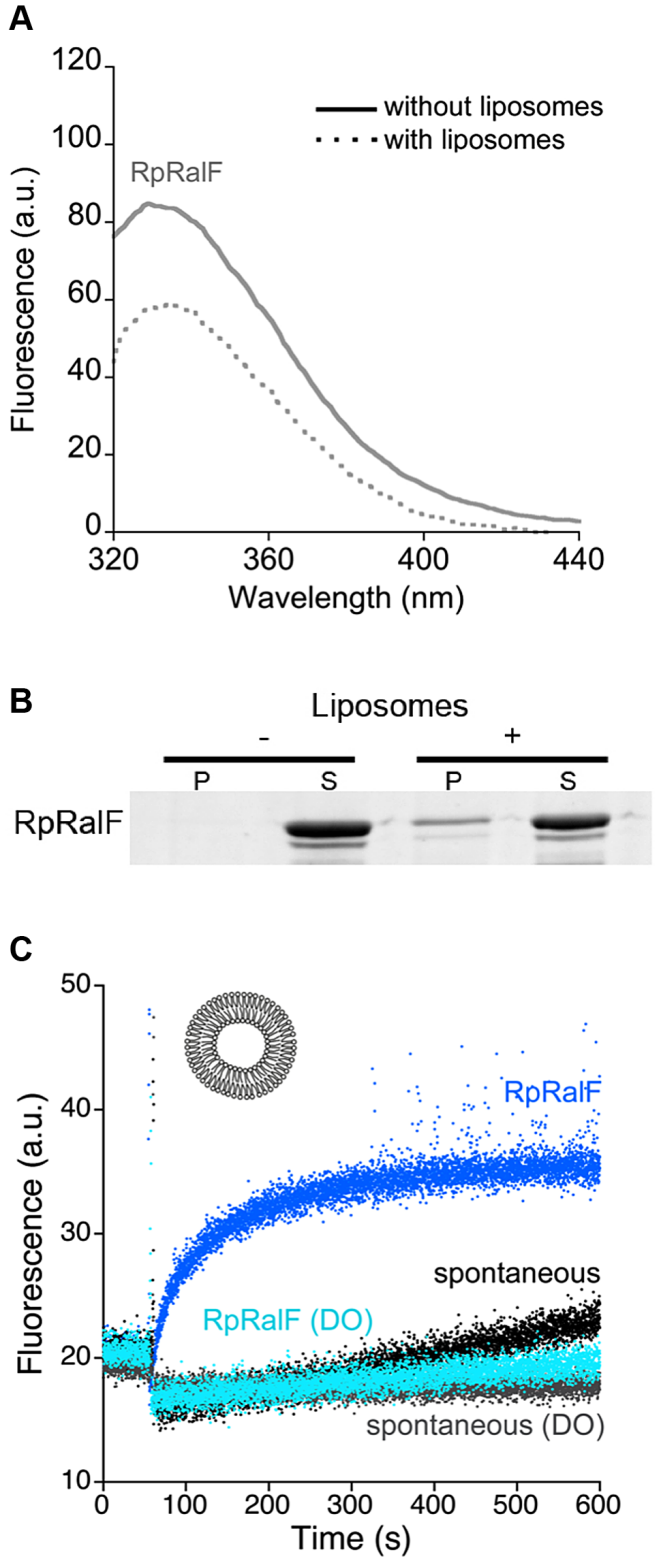
*Rickettsia prowazekii* RalF is activated by membranes. A. Interaction of RpRalF with liposomes analyzed by tryptophan fluorescence. Fluorescence scans done without and with liposomes are in plain and dotted lines, respectively. **B.** **Co-sedimentation of RpRalF with liposomes.** P = pellet, S = supernatant. **C.** **Representative nucleotide exchange kinetics of ^myr^Arf1 activation by RpRalF in the presence of liposomes.** Experiments were carried out with ^myr^Arf1 (0.4 µM) and RpRalF (0.1 µM) and 200 µM liposomes. Experiments carried out with liposomes containing dioleoyl lipids extruded at 0.4 µM (composition as in **Figure 2A**) are labeled DO. Unless indicated otherwise, the composition of liposomes is as in **Figure 1D**.

Several species of *Rickettsia* encode a RalF homolog of unknown function, and these RalF proteins all have a conserved capping domain that includes an aromatic cluster (**Figure S3**). Unlike *Legionella*, *Rickettsia* do not replicate in an intracellular vacuole (reviewed in [12]), raising the issue of whether *Rickettsia* RalF proteins function as membrane-regulated ArfGEFs. Purified *Rickettsia prowazekii* RalF (RpRalF) (**Figure S1A**) displayed a marked decrease of tryptophan fluorescence upon addition of liposomes (**Figure 3A**), indicating that membranes modify the environment of the unique tryptophan in the aromatic cluster, Trp283. Direct interaction of RpRalF with membranes was further confirmed by its co-sedimentation with liposomes (**Figure 3B**). To analyze whether the mechanism of activation by membranes observed for LpRalF also applies to RpRalF, we compared its GEF activity in solution and in the presence of membranes. RpRalF was strongly autoinhibited in solution (k_obs_ = 4±1.2 10^−4^ s^−1^ measured at 1 µM RpRalF and 1 µM Δ17Arf1, which is very close to the k_obs_ = 8±3.5 10^−4^ s^−1^ measured in the absence of GEF). Liposomes containing anionic lipids stimulated nucleotide exchange on ^myr^Arf1 at a level similar to that observed for LpRalF (k_cat_/K_m_ = 2.85±0.09 10^5^ M^−1^s^−1^, **Figure 3C**). Thus, RpRalF is a membrane-interacting ArfGEF that is auto-inhibited in solution and strongly activated by membranes. Given the sequence conservation between LpRalF and RpRalF, these data suggest that RpRalF similarly uses its capping domain for both auto-inhibition and membrane binding, despite the different intracellular lifestyles of these two pathogens. However, RpRalF was not activated by liposomes containing dioleoyl lipids (**Figure 3C**), in striking contrast with LpRalF under the same conditions, indicating that they have different lipid preferences.

### The aromatic cluster of LpRalF capping domain fine-tunes the timing of Arf activation on the LCV

**Figure 4:**
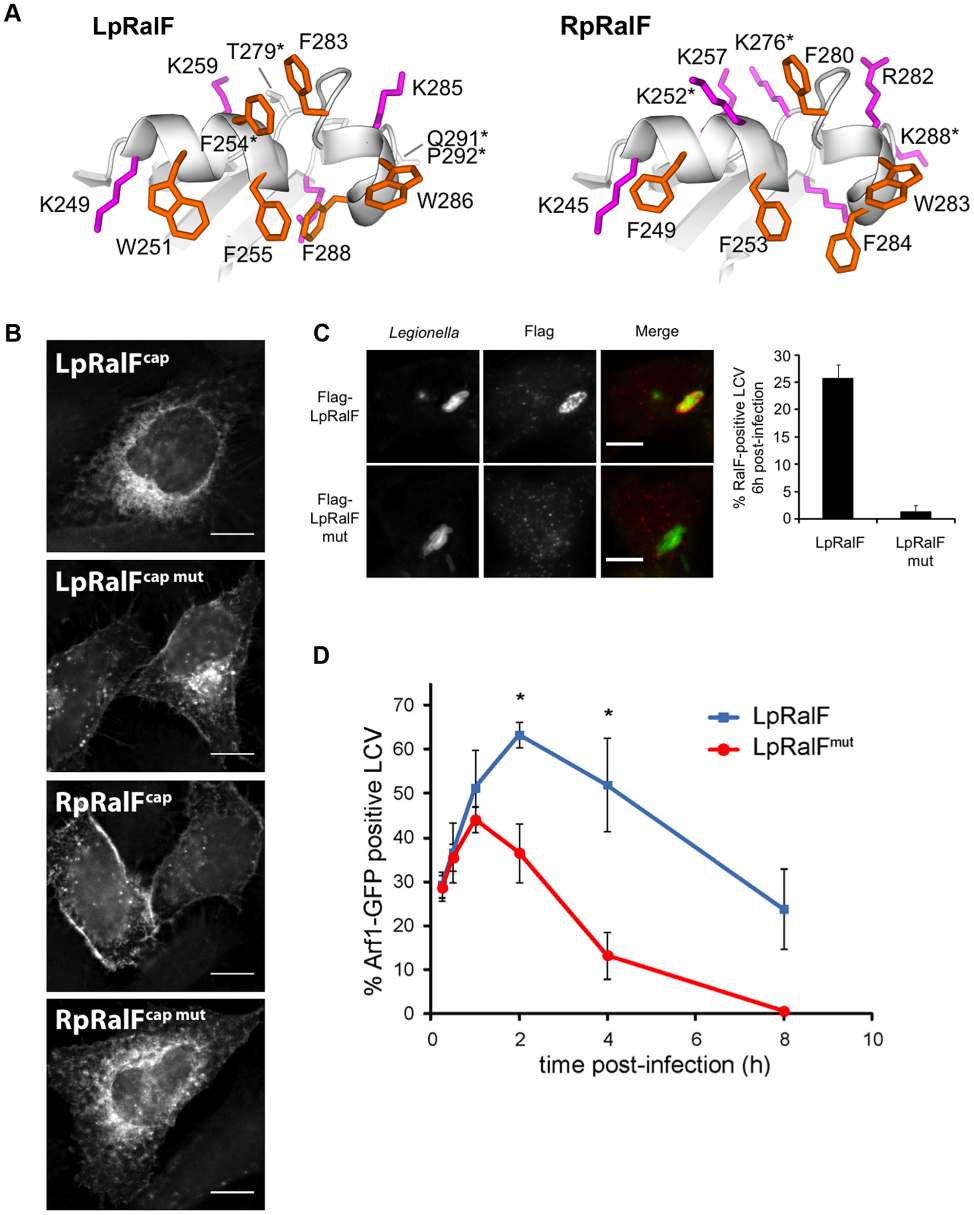
The aromatic cluster determines the capping domain localization to specific intracellular membranes. A. The aromatic clusters of LpRalF (left) and RpRalF (right) have a different ratio of aromatic (orange) and positively charged (magenta) residues. RpRalF was modeled from the crystal structure of LpRalF (PDB entry 1XSZ, [13]). Residues mutated in the following experiments are indicated by an asterisk. **B.** **The aromatic cluster determines LpRalF and RpRalF capping domain localization.** HeLa cells expressing the indicated YFP-tagged RalF constructs were examined by fluorescence microscopy. Bar = 10 µm. **C.** **Mutations in the aromatic cluster alter the residence time of LpRalF at the LCV.** HEK293 cells were infected with *L. pneumophila ΔralF* complemented with a plasmid encoding either 3*Flag-LpRalF or 3*Flag-LpRalF^mut^. 6 h post-infection, cells were fixed and stained with anti-Flag antibodies (red). *Legionella* were stained with anti-*Legionella* antibodies (green). Bar = 10 µm. Quantification of RalF-positive vacuoles is shown. Represented is the average ± SEM (standard error of the mean) of 3 experiments where 50 vacuoles were counted. **D.** **Mutations in the aromatic cluster of LpRalF alter the kinetics of Arf1 recruitment at the LCV.** HEK293 cells stably expressing Arf1-GFP were infected with *L. pneumophila ΔralF* complemented with a plasmid encoding either 3*Flag-LpRalF or 3*Flag-LpRalF^mut^. Arf1-GFP recruitment to the LCV was quantified at different time points post-infection. RalF and RalF^mut^ were expressed, translocated and present in the host cell 6 hours post-infection at the same level. Represented is the average ± SEM of 3 experiments where 50 vacuoles per time point were counted. (* P<0.05).

Our *in vitro* analysis predicts that LpRalF uses the membrane sensor encoded in the aromatic cluster to spatially and temporally regulate Arf activation during the course of LCV maturation. As a first indication, we observed that the F255K mutation in the aromatic cluster was sufficient to displace LpRalF capping domain expressed in HeLa cells (YFP-LpRalF^cap^) from the reticulate perinuclear pattern reminiscent of the ER observed for wild-type YFP-LpRalF^cap^ [14] to a Golgi pattern (**Figure S2E**). Consistently, despite its high exchange activity on negatively charged liposomes *in vitro*, M45-flagged LpRalF^F255K^ failed to recruit Arf1 at the LCV in infected cells, likely due to mislocalization. Interestingly, the capping domains of LpRalF and RpRalF have strikingly divergent localizations when expressed in eukaryotic cells [14] and have different ratio in aromatic and positively charged residues in their aromatic clusters (**Figure 4A**). Because the aromatic cluster is involved in membrane sensing, divergences between LpRalF and RpRalF aromatic clusters could explain the different localizations of the two capping domains. To test this hypothesis, we introduced reciprocal mutations in LpRalF and RpRalF capping domains (F254K, T279K, Q291K and P292S in LpRalF^cap^; K252F, K276T and K288Q in RpRalF^cap^). Recombinant MBP-LpRalF^capmut^ bound to negatively charged lipids in a lipid overlay assay, unlike MBP-LpRalF^cap^ but reminiscent of MBP-RpRalF^cap^ (**Figure S4A, top**). Conversely, MBP-RpRalF^capmut^ lost the ability of MBP-RpRalF^cap^ to bind to negatively charged lipids in this assay (**Figure S4B, bottom**). When expressed in HeLa cells, YFP-LpRalF^capmut^ relocated from the perinuclear localization observed for YFP-LpRalF^cap^ to the plasma membrane and to Golgi-like structures (**Figure 4B** **, top**). Conversely, YFP-RpRalF^capmut^ relocated from the plasma membrane to a perinuclear localization (**Figure 4B** **, bottom**). These data indicate that the content in aromatic and positively charged residues in the aromatic cluster is a major determinant of the subcellular localization of the capping domain.

To analyze whether the aromatic cluster controls the localization of the full-length RalF protein and the timing of Arf1 activation during the maturation of the LCV, we infected cells with *L. pneumophila ΔralF* expressing either 3*Flag-LpRalF or 3*Flag-LpRalF^mut^ carrying the F254K, T279K, Q291K and P292S mutations. Both constructs were expressed and translocated to similar levels (**Figures S4B and S4C**). Strikingly, while LpRalF was still present at the surface of a large fraction of LCVs at 6 h post-infection (26%±2.4), LpRalF^mut^ could not be detected (1.3%±1.15) (**Figure 4C**), indicating that the aromatic cluster controls LpRalF localization. Next, we compared the timing of Arf1 activation on the LCV (**Figure 4D**). Both LpRalF constructs were equally efficient at recruiting Arf1 one hour after infection. However, while Arf1 activation by LpRalF continued to rise for another hour and was still detectable after 8 hours, its activation by LpRalF^mut^ decreased after one hour and became undetectable after 4 hours. Altogether, these data indicate that the aromatic cluster functions as a membrane sensor *in vivo* and that it drives the timing of Arf1 activation during infection in accordance with the ratio between aromatic and positively charged residues.

### The capping domain is involved in the intramolecular organization of active LpRalF

**Figure 5:**
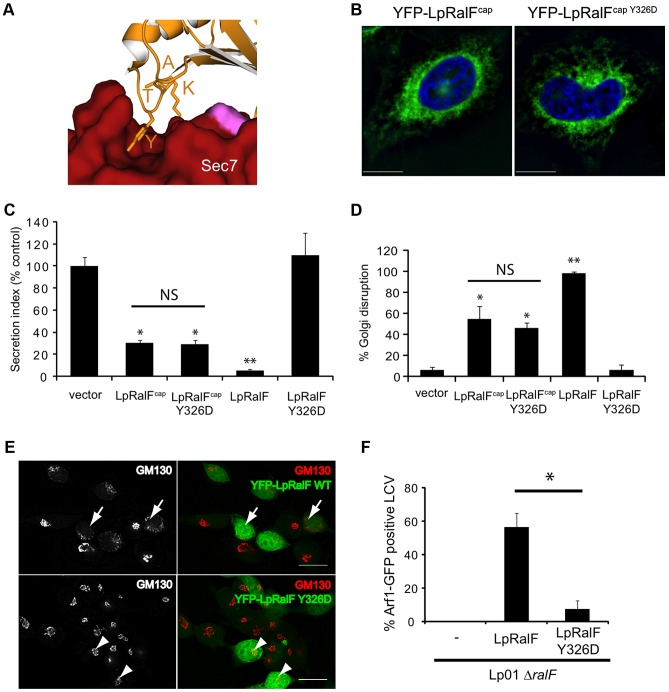
LpRalF^Y326D^ cannot reach the active conformation. **A.** **A close-up view of the structure of the conserved KATY motif** from the capping domain in interaction with the N-terminus of the Sec7 domain. **B.** **The Y326D mutation does not alter the localization of the capping domain.** HeLa cells were transfected with plasmids expressing YFP-LpRalF^cap^ or YFP-LpRalF^capY326D^. Cells were fixed 24 h after transfection and visualized by fluorescence microscopy. DAPI staining is shown in blue. Bar = 10 µm. **C-D-E.** **The Y326D mutation disrupts the anti-secretion and Golgi disruption activities of full-length LpRalF but not of its capping domain.** **C-** Measurement of alkaline phosphatase secretion in HEK293 cells expressing YFP or the indicated YFP-LpRalF constructs. The results are normalized so the cells expressing the empty plasmid have a secretion value of 100%. These data are representative of 3 independent experiments done in triplicate (* P<0.05, * P<0.005). **D and E-** Golgi disruption was quantified in HeLa cells expressing YFP or the indicated YFP-LpRalF constructs. Cells were fixed 24 h after transfection and stained for GM130. Quantification represents the average of 3 independent experiments where 50 cells were counted. Standard deviations are indicated (* P<0.05, ** P<0.005). Representative experiments are shown in **Panel E.** Arrows indicate disrupted Golgi, arrowheads indicate intact Golgi. **F.** **LpRalF^Y326D^ does not complement LpRalF for Arf1 recruitment at the LCV.** HEK293 cells stably expressing Arf1-GFP were infected with *L. pneumophila* Δ*ralF* complemented with M45-LpRalF or M45-LpRalF^Y326D^. Arf1-GFP recruitment to the LCV was quantified 1 h post-infection. Represented is the average of 3 independent experiments where 50 vacuoles were counted. Standard deviations are indicated (* P<0.01).

In addition to blocking the Sec7 catalytic site by its aromatic cluster, the capping domain is also involved in intramolecular interactions with the N-terminus of the Sec7 domain that stabilize the auto-inhibited conformation in LpRalF [13] (**Figure 1A**). The contact is mediated by a 4-residue motif found in all RalF homologs, ^323^KATY^326^ in LpRalF, of which the conserved tyrosine forms a direct interaction with the Sec7 domain (**Figures 5A** **and S3**). To investigate the contribution of this motif to RalF regulation and function, we characterized LpRalF constructs carrying a Y326D mutation. We first analyzed the properties of the capping domain carrying this mutation. YFP-LpRalF^capY326D^ ectopically expressed in HeLa cells showed a perinuclear localization phenotype that was similar to wild-type YFP-LpRalF^cap^ (**Figure 5B**), which was in contrast to capping domain proteins having mutations in the aromatic cluster (compare with **Figures 4B** **and S2E**). Likewise, YFP-LpRalF^capY326D^ retained the ability to impair secretion (**Figure 5C**) and to disrupt the Golgi (**Figure 5D**) previously observed for wild-type YFP-LpRalF^cap^ [14]. Full-length YFP-LpRalF^Y326D^ ectopically expressed in HeLa cells, however, did not impair secretion (**Figure 5C**) or disrupt Golgi architecture (**Figures 5D and 5E**), unlike full-length wild-type YFP-LpRalF. In addition, recombinant full-length LpRalF^Y326D^ was unable to activate Arf1 in the presence of liposomes (k_obs_ = 9±1.2 10^−5^ s^−1^ at 0.4 µM ^myr^Arf1 and 0.2 µM LpRalF^Y326D^, to be compared to 4.7±1.0 10^−2^ s^−1^ for LpRalF under the same conditions) and *Legionella ΔralF* expressing full-length M45-tagged LpRalF^Y326D^ failed to recruit Arf1 at the LCV during infection (**Figure 5F**). These observations indicate that the mutation is silent in the context of the capping domain alone but is inactivating in the context of the full-length protein, which suggests that the KATY motif is necessary for reorganization of LpRalF to an active conformation.

## Discussion

Previous studies showed that the ArfGEF activity of *Legionella* RalF is auto-inhibited in solution, which implied that there must be a cellular mechanism for activation that remained unknown [13]. In this work, we demonstrate that interactions between an aromatic cluster of amino acids in the LpRalF C-terminal capping domain and lipid membranes provides a signal that relieves autoinhibition and converts LpRalF to a highly potent ArfGEF. Auto-inhibition and membrane recruitment are mediated by the same region of the capping domain, thus making both states mutually exclusive and establishing a membrane-driven binary switch. Thus, *Legionella* has evolved a minimal version of the regulatory mechanisms found in eukaryotic GEFs, which enables it to by-pass the cellular regulation of the small GTPase Arf by a single functional site that derepresses the ArfGEF activity when RalF encounters a favorable lipid environment.

Our data show that the membrane sensor of the capping domain of LpRalF contains protruding α-helices that expose aromatic and lysine residues. This atypical composition and structure endows it with a dual sensitivity to packing and electrostatic properties of lipid membranes, but not to curvature. We propose that the aromatic residues encode the sensitivity of LpRalF to packing defects by peripheral insertion into the lipid bilayer, and the lysines recognize negatively charged membrane surfaces by electrostatic interactions. The absence of a recognizable pocket that could accommodate lipid polar headgroups suggests that the recognition of negatively charged lipids is largely non-specific. The highly convex shape of the sensor might also explain why it does not detect membrane curvature. These characteristics make the capping domain a unique membrane-binding determinant with combined membrane sensitivities that depart from those of specialized phosphoinositide-binding domains, such as PH or FYVE domains (reviewed in [25]), or those of curvature-sensing domains, such as ALPS or BAR domains (reviewed in [26]).

**Figure 6:**
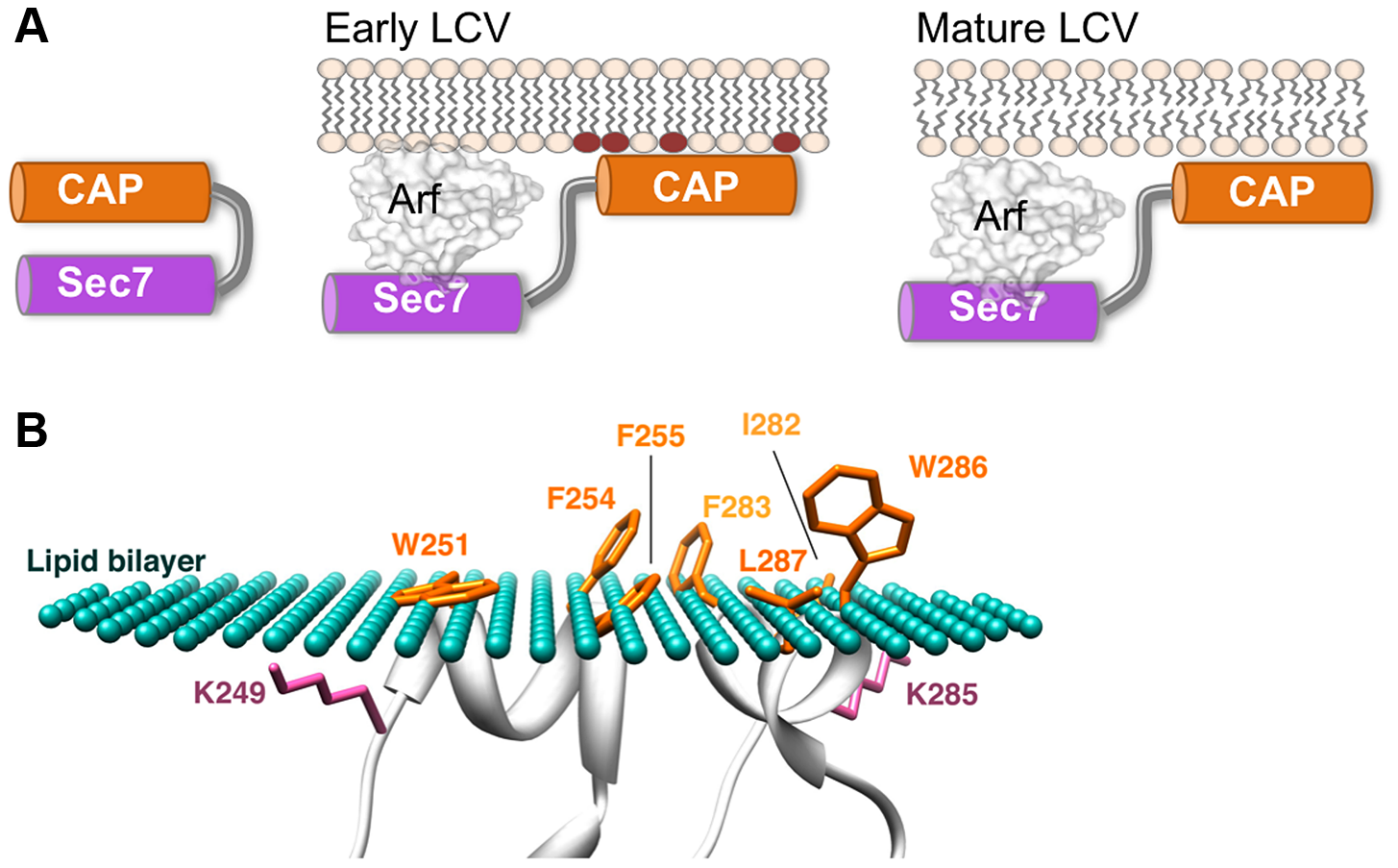
A model of LpRalF peripheral recruitment and activation on membranes. **A.** **A model of LpRalF activation** by negatively charged lipids of the plasma membrane-derived early LCV (phospholipid headgroups shown in brown) and sustained activation on less packed and less charged ER-like membranes of the mature LCV. **B.** **A model of peripheral insertion of the aromatic cluster into membranes.** The membrane surface is depicted as green dots and membrane-embedded residues are in orange. The calculated maximal penetration depth of LpRalF^cap^ atoms into the lipid hydrocarbon core is 5.2±1.0 Å. Modeling was done with the PPM server (http://opm.phar.umich.edu/).

Why would LpRalF have evolved to utilize a dual sensitivity membrane sensor? LpRalF is injected rapidly during uptake of *Legionella* and the ArfGEF activity promotes recruitment of Arf1 to the nascent LCV [13]. LpRalF remains on the LCV after internalization of the bacteria [4]. A major feature of the LCV is that it rapidly converts from a plasma membrane-derived to an ER-like organelle [3], [6]. Accordingly, the LCV is predicted to rapidly loose the negatively charged character of the plasma membrane, and to acquire the characteristics of ER membranes, which are not enriched for negatively charged lipids and are more loosely packed (reviewed in [26]). We propose that the ability of the capping domain to sense these unrelated membrane environments allows LpRalF to be activated soon after its injection on the nascent LCV and to remain attached as the LCV maturates by incorporating ER vesicles (**Figure 6A**). Hence, the capping domain would directly monitor the duration of Arf activation on the LCV.

Our results strongly suggest that activation of *Rickettsia* RalF also requires activation through displacement of the capping domain resulting from its recruitment to membranes, according to a scenario similar to that demonstrated in this study for *Legionella* RalF. However, *R. prowazekii* is phylogenetically distant from *Legionella* species and rapidly escapes from the phagosome to replicate in the cytosol (reviewed in [12]). We speculate that the RalF protein family is comprised of xenologs, meaning that an ancestral *ralF* gene was transmitted between different bacterial species though horizontal gene transfer and this gene then evolved divergently to activate cellular Arf proteins on distinct subcellular membranes. The observation that variations in the aromatic and positively charged residues in the membrane sensor modulate membrane targeting properties suggests that the sensor probably also plays a critical role in *Rickettsia* infection by restricting RpRalF to specific membranes. The difference in lipid preference between RpRalF and LpRalF and the combined ability of the capping domain of RpRalF to target the plasma membrane and to interfere with the actin cytoskeleton [14] suggest that RpRalF could be involved in bacterial entry into host cell or that it could control actin-dependent processes at the plasma membrane after uptake, thus representing a remarkable case where similar biochemical activity results in unique effects.

The active conformation of RalF proteins has not been resolved structurally. Our analysis suggests that membranes are mandatory to stabilize this open conformation. However, our data provide indirect insight into the nature of the conformational change that are required for derepression of the ArfGEF activity. First, the aromatic cluster should face the membranes, which can be modeled with the twin helices parallel to the membrane (**Figure 6B**) [21]. Second, the aromatic cluster in membrane-bound RalF should lie in the same plane as the myristoylated N-terminal helix of Arf, so that both can interact with membranes simultaneously. The position of Arf1 bound to RalF^Sec7^ can be modeled from the crystal structures of Arf/Sec7 complexes (**Figure 1A**) [22], [23], suggesting that the capping domain should swing by at least 90 degrees to release autoinhibition. The short length of the linker that connects the Sec7 and capping domains (^191^PFELNFVKTSP^201^ in LpRalF, **Figure 1A**) and our analysis of the role of the KATY motif in supporting the organization of active LpRalF raises the possibility that the capping domains remains in contact with the N-terminus of the Sec7 domain throughout the exchange reaction, as seen in BRAG ArfGEFs [27]. Structural elucidation of the Arf/RalF complex, which escaped biochemical isolation so far, is now needed to unravel the details of this mechanism.

## Materials and Methods

### Strains

*Legionella pneumophila* serogroup 1, strain Lp01 [28], and the *ΔralF* mutant [4] were used for infection experiments. *Legionella* strains were grown on charcoal yeast extract (CYE) plates (1% yeast extract, 1% *N*-(2-acetamido)-2-aminoethanesulfonic acid (ACES; pH 6.9), 3.3 mM l-cysteine, 0.33 mM Fe(NO_3_)_3_, 1.5% bacto-agar, 0.2% activated charcoal), supplemented with 10 µg/mL chloramphenicol when required [29].

### Cloning, proteins production and purification

For *in vitro* experiments, *Legionella pneumophila* RalF was subcloned into the pHis-1 vector [13] and used as a matrix for the mutants using the QuickChange site-directed mutagenesis kit (Stratagene). The sequence coding for the Sec7 domain (residues 1 to 201) of LpRalF was PCR amplified and cloned into the Gateway destination vector pDEST14 (Invitrogen). The codon optimized RpRalF sequence (Genescript) was subcloned in the pDEST17 vector (Invitrogen). All clones were confirmed by sequencing (GATC Biotech).

MBP-tagged LpRalF195–374 and RpRalF189–359 were cloned in pMALc5x vector (BamHI/EcoRI). LpRalF1–374, LpRalF192–374 and RpRalF189–359 were subcloned in pYFPC1 (EcoRI/BamHI) for expression in eukaryotic cells. For expression in *L. pneumophila*, LpRalF was subcloned in pJB1806 (M45-tagged) or pSN85 (3*Flag-tagged) vectors (*BamHI*/*SalI*). Site-directed mutagenesis was performed to obtain single point mutants. Plasmids were amplified using two complementary primers containing the desired mutation with Pfu turbo (Stratagene). The product was digested by *DpnI* for 1 h at 37°C before transformation in DH5α. MBP-RalF constructs were purified as in [14].

All other recombinant proteins were produced in BL21(DE3)Star or Rosetta(DE3)pLysS *Escherichia coli* strains in 2×TY medium by inducing with 0.5 mM IPTG at 20°C. After centrifugation at 5000 g for 30 minutes, bacterial pellets were resuspended in 40 mL of lysis buffer (50 mM Tris pH 8.0, 300 mM NaCl, 10 mM imidazole, 0.25 mg.mL^−1^ lysozyme, anti-proteases, except for RpRalF for which Tris pH 8.0 was replaced by Tris pH 9.0 and 600 mM NaCl was used instead of 300 mM) per liter of culture and frozen at −80°C. After thawing, cells were sonicated, cleared by centrifugation at 20 000 g for 30 minutes and the supernatant was filtered over a 0.22 µm filter. Proteins were purified by an affinity step on a 5 mL HisTrap nickel affinity column (GE Healthcare) using 60 mM imidazole for elution, followed by gel filtration on a Superdex 200 column (GE Healthcare) in a buffer containing 10 mM Tris pH 8.0, 150 mM NaCl, and 2 mM β-mercaptoethanol. Protein purity was confirmed by SDS-PAGE, and all proteins were well folded as assessed by circular dichroism, allowing for their accurate kinetics analysis. Purified proteins were concentrated to at least 10 mg.mL^−1^ before storing at −80°C with 10% glycerol. Human Arf1 truncated of its N-terminal helix (Δ17Arf1) was expressed and purified as described in [23], and was loaded with GDP prior to all experiments. Myristoylated Arf1 (^myr^Arf1) was obtained by co-expression of yeast myristoyltransferase and purified as described in [30]. ^Myr^Arf1 expressed in bacteria is readily fully loaded with GDP.

### Reconstitution of LpRalF^Sec7^/Arf complexes

Formation of the complexes between LpRalF^Sec7^ constructs and Δ17Arf1 was analyzed by gel filtration on a Superdex 75 10/300GL (GE Healthcare) and visualized by SDS-PAGE. For the nucleotide-free complex, LpRalF^Sec7^ (residues 1–201) was incubated with an excess of Δ17Arf1 and incubated with agarose bead-coupled alkaline phosphatase (Sigma Aldrich) for 12 hours at 4°C, followed by centrifugation at 16000 g to remove the beads. The complex between Δ17Arf1-GDP and LpRalF^Sec7/E103K^ was obtained by incubation in 10 mM Tris pH 8.0, 150 mM NaCl, 2 mM β-mercaptoethanol, 2 mM EDTA, 1 mM MgCl_2_ for 10 minutes at room temperature as described [23], [31].

### Liposomes preparation

All lipids were from Avanti Polar Lipids. Liposomes were prepared as described in [32] and freshly extruded on 0.03, 0.1, or 0.4 µm filters as indicated. Extruded liposomes were stored at room temperature and used within two days.

### Nucleotide exchange fluorescence kinetics assays

Tryptophan fluorescence (λ_exc_ = 290 nm, λ_em_ = 340 nm) was used to follow GDP to GTP exchange as described [32]. All fluorescence measurements were performed using a Varian Carry Eclipse fluorimeter. Samples (800 µL) were thermostated at 37°C and continuously stirred. Nucleotide exchange kinetics in solution were measured with Δ17Arf1-GDP (1 µM) in 50 mM Tris pH 8.0, 50 mM NaCl, 2 mM β-mercaptoethanol, with RalF constructs concentrations in the 0.5–10 µM range for k_cat_/K_m_ determination, or 1 µM for single k_obs_ measurements. Nucleotide exchange assays with liposomes were done with ^myr^Arf1 (0.4 µM) in the presence of 200 µM liposomes, in 50 mM Hepes pH 7.4, 120 mM potassium acetate, 1 mM MgCl_2_ (HKM buffer) with RalF concentrations in the 0.05–0.3 µM range for k_cat_/K_m_ determinations, or 0.1 µM for single k_obs_ measurements. Nucleotide exchange was triggered by addition of 100 µM GTP. Activation rate constants (k_obs_, s^−1^) were determined by fitting the fluorescence changes to a single exponential using Kaleidagraph software. The catalytic efficiency k_cat_/K_m_ (M^−1^s^−1^) was determined by linear fitting of k_obs_ values as a function of the GEF concentration (in M). All experiments were done at least in triplicate.

### Membrane-binding experiments

For the immunogold electron microscopy analysis, 100 nM of His-tagged LpRalF was incubated with 50 µM extruded liposomes for 5 min in HKM buffer. Samples were applied to carbon-coated 400 mesh Nickel grids (Agar Scientific) for 1 min and subsequently blocked with HKM buffer containing 1% BSA for 45 min. The grids were then incubated for 1.5 hours with a mouse anti-His antibody (Qiagen) at a dilution of 1∶200. After batch washing with HKM buffer, the grids were incubated with 10 nm colloidal gold anti-mouse secondary antibody (Agar Scientific) for 1.5 hours and batch washed with HKM buffer. The grids were then stained with 2% uranyl acetate for 30 seconds. Images were acquired on a JEOL 1400 transmission electron microscope using low-dose conditions at 120 kV with a tungsten filament. Images were recorded using a Gatan 4k×4k CCD camera. We controlled that negatively stained grids containing only soluble RalF were labeled, but not grids containing only liposomes, indicating that immunogold labeling shown in Figure 1C is specific of liposome-bound RalF.

For the tryptophan fluorescence experiments, 2 µM RalF or RalF mutants was incubated with 500 µM liposomes in HKM buffer at 37°C under stirring prior to excitation at 297.5 nm. Tryptophan fluorescence scans were recorded for the buffer without or with liposomes, and were substracted from the scans recorded in the presence of wild type or mutant RalF.

Liposome sedimentation assays were done with RalF proteins (2 µM) incubated in 50 mM Tris pH 7.5, 120 mM NaCl, 1 mM MgCl_2_, 1 mM DTT for 10 min at room temperature with sucrose loaded fluorescent liposomes (39% PC, 20% NBD-PE, 25% PS, 1% PIP2, 15% cholesterol) extruded on 0.4 µm filter. After centrifugation for 20 min at 400000 g, liposome sedimentation was checked and quantified by fluorescence using a Fuji BioImager equipped with a CCD camera. Pellets were loaded on a 15% SDS-PAGE. Proteins were stained with Sypro-orange.

### Membrane strip assay

LpRalF and RpRalF capping domains affinity for lipids was assessed using commercially available membrane lipid strips (Echelon) as described in [14]. Briefly, membranes were incubated for 1 h at room temperature with purified MBP-tagged RalF, and the binding was visualized by chemiluminescence using anti-MBP antibodies.

### Crystallographic, SAXS and modeling analysis

Crystals of LpRalF^F255K^ mutant were obtained by vapor diffusion in 0.1 M Hepes pH 7–8, 20–30%, PEG1000, 1.5 mM Fos-choline-12 and cryoprotected with a mix of parafin and silicon oils (50∶50 v∶v). A complete diffraction dataset at 3.1 Å resolution was collected at the Proxima-1 beamline (SOLEIL synchrotron, Gif-sur-Yvette, France) and integrated with the program XDS [33]. Crystals belong to space group P3_1_21, with one molecule per asymmetric unit. The structure was solved by molecular replacement with the program Phaser [34] using wild-type *Legionella* RalF as a model (PDB entry 1XSZ, [13]). Refinement was carried out with the program BUSTER [35], in alternation with graphical building using Coot [36]. Data collection and refinement statistics are reported in **Table S1**. Atomic coordinates and structure factors have been deposited with the Protein Data Bank with accession code 4c7p. SAXS experiments were conducted on beamline SWING (SOLEIL Synchrotron, Gif-sur-Yvette, France) essentially as described in [37]. The histidine tags were cleaved to avoid noise in the SAXS data. Samples were prepared in 10 mM Tris pH 8.0, 150 mM NaCl, 2 mM β-mercaptoethanol, centrifuged at 16100 g for 30 min before the SAXS experiment and used at four concentrations (10, 5, 2.5 and 1.25 mg.mL^−1^). SAXS data were reduced with FOXTROT and analyzed with the ATSAS suite (EMBL, Hamburg, www.embl-hamburg.de/biosaxs/software.html). Modeling of LpRalF peripheral insertion in membranes was done using the PPM server (http://opm.phar.umich.edu/server.php) [38].

### *Legionella* infections

*Legionella* were harvested from 2-day heavy patch, and used to infect HEK293 cells stably expressing Arf1-GFP and the receptor Fcγ. This receptor allows *L. pneumophila* opsonized with anti-*Legionella* antibodies to be internalized efficiently by non-phagocytic cells [39]. Bacteria were opsonized with rabbit anti-*Legionella* antibody diluted 1/1000 for 30 min at 37°C. Bacteria were then added to the cells at a multiplicity of infection of 1. The cells were centrifuged 5 min at 1000 rpm and incubated at 37°C. Cells were then fixed with PFA for 20 min at room temperature, and stained for extracellular bacteria with blue anti-rabbit antibodies. Permeabilization was performed by treatment with cold methanol 1 min at RT before staining total bacteria with red anti-rabbit antibodies. The number of LCVs positive for Arf1-GFP was quantified. For kinetics assays of Arf1 recruitment to the LCV, we controlled that RalF and RalF^mut^ were expressed, translocated and present in the host cell 6 hours post-infection at the same level.

### Cell culture, transient transfections and cell imaging

Human Embryonic Kidney cells (HEK293) and HeLa cells were maintained in minimal Dulbecco's Modified Eagle's Medium, supplemented with 10% heat inactivated fetal bovine serum, 100 µg.mL^−1^ penicillin and 10 µg.mL^−1^ streptomycin at 37°C with 5% CO_2_. For transfection, Hela cells were plated at a density of 10^5^ cells per well in 24-well tissue culture plates with glass coverslips and transfected the following day using effectene reagent (Qiagen). After transfection, cells were incubated for 24 hours then fixed with 3% PFA for 20 min at room temperature. Cells were permeabilized in Blocking Buffer (0.2% saponin, 0.5% BSA, 1% fetal calf serum in PBS) for 20 min. Coverslips were then washed with PBS and incubated with mouse anti-GM130 (BD Biosciences, diluted 1/1000 in Blocking Buffer) for 1 h at room temperature. Coverslips were then washed with PBS and incubated with anti-mouse TexasRed conjugated antibody at a dilution of 1/250 in Blocking Buffer for 1 h at room temperature. Finally, cells were washed in PBS and mounted on plain microscope slides. Cells were subsequently visualized by fluorescence microscopy using a Nikon Eclipse TE2000-S microscope and a 100×/1.40 oil objective (Nikon Plan Apo). Z-stacks were acquired using a Hamamatsu ORCA-ER camera and 3D max was generated. Images were exported to Image J and deconvoluted for the production of figures.

### Secretion assay

HEK293 cells were plated in 24-well dishes at a density of 3.10^4^ cells per well. After 18 hours incubation, cells were cotransfected with 200 ng of plasmid encoding the indicated YFP-tagged protein and 300 ng of a plasmid encoding a secreted alkaline phosphatase (SEAP) protein. 24 hours after transfection, cells were washed, and fresh tissue culture medium was added. SEAP activity was measured 7 hours later, in the supernatant and in cells, using the Phosphalight SEAP kit (Applied Biosystems). The ratio of SEAP activity detected in the culture medium to the cells-associated SEAP activity is measured. Data are then normalized and compared to control cells, expressed as percent of control cell activity.

## Supporting Information

Figure S1 **Characterization of constructs used in this study.** **A.** **SDS-PAGE analysis of LpRalF and RpRalF constructs used in this study.** **B.** **LpRalF^Sec7 E103K^ forms a stable complex with Arf1-GDP.** The size exclusion chromatography profile of Arf1-GDP alone is in red, of LpRalF alone in green, and of Arf1 and LpRalF in blue. Below: SDS-PAGE analysis of the Arf1/LpRalF^Sec7 E103K^ experiment. **C.** **LpRalF^Sec7^ forms a stable complex with nucleotide-free Arf1.** Color coding and analysis are as in **Figure S1B**. **D.** **LpRalF is not regulated by a feed-back loop.** Nucleotide exchange kinetics were analyzed in the presence of the Arf1-binding region of the Arf effector GRAB, which should deplete Arf1-GTP as it is produced by RalF. The nucleotide exchange experiment was carried out with liposomes (200 µM), ^myr^Arf1-GDP (0.4 µM), LpRalF (0.1 µM) with or without addition of GRAB.
(TIF)

Figure S2 **The aromatic cluster of** ***Legionella pneumophila*** **RalF is a membrane sensor.** **A–B.** **LpRalF is sensitive to liposome composition and packing defects but not to curvature.** Representative fluorescence kinetics of Arf1 activation using liposomes of indicated compositions and curvatures. All experiments were carried out with liposomes (200 µM) extruded through a 0.1 µm filter unless indicated otherwise, ^myr^Arf1-GDP (0.4 µM), LpRalF (0.1 µM) and were started with 40 µM GTP. **C.** **The crystal structure of LpRalF^F255K^ retains the auto-inhibited conformation.** The location of aromatic cluster is shown in magenta for LpRalF^F255K^, in red for LpRalF. **D.** **Representative fluorescence kinetics of the F255 LpRalF mutants in the presence of liposomes.** Experiments were done with 0.05 µM LpRalF, 0.4 µM ^myr^Arf1 and 200 µM liposomes (composition as in **Figure 1D**). **E.** **Localization of LpRalF and LpRalF^F255K^ capping domains when ectopically expressed in HeLa cells.** Cells transfected with YFP-LpRalF^cap^ or YFP-LpRalF^capF255K^ were fixed 24 hours after transfection, stained with anti-GM130 antibodies (red) and DAPI (blue) and analyzed by fluorescence microscopy. Bar = 5 µm.
(TIF)

Figure S3 **Alignment of RalF sequences from various** ***Legionella*** **and** ***Rickettsia*** **species.** Residues in LpRalF permuted to the corresponding RpRalF residues are labelled with *. Residues in RpRalF permuted to the corresponding LpRalF residues are labelled with #. Done with MultAlin and drawn with ESPript.
(TIF)

Figure S4 **Characterization of LpRalF and RpRalF mutants.** **A.** **Alignment of LpRalF and RpRalF α-helices forming the aromatic cluster and lipid overlay assay.** Divergent residues mutated in LpRalF (top) and RpRalF (bottom) in the subsequent experiments are shown with a red square. A protein-lipid overlay assay shows that mutations in the aromatic cluster modify lipid-binding properties of LpRalF (A) and RpRalF (B) capping domains. The binding of wild-type and mutants MBP-tagged capping domain to indicated lipids immobilized on nitrocellulose membranes was analyzed using an anti-MBP antibody. **B.** **Expression of tagged proteins used to complement Lp01** ***ΔralF*** **.** Western blot on *L. pneumophila* Lp01 crude extracts expressing different constructs used in this study. **C.** **Similar translocation of LpRalF and LpRalF^mut^ by** ***Legionella*** **type IV secretion system.** HEK293-FcγRII cells were infected with *L. pneumophila* wt or *ΔdotA* carrying a plasmid encoding the indicated Cya fusion proteins. cAMP level in the cell cytosol was quantified 1 h post-infection. Data are mean ± SD from three independent samples.
(TIF)

Table S1 **Data collection and refinement statistics of the LpRalF^F255K^ mutant crystal structure.**
(DOCX)
