# Supplementary figures and images for: A Novel Membrane Sensor Controls the Localization and ArfGEF Activity of Bacterial RalF
Source: PLoS Pathog. 2013 Nov 14;9(11):e1003747. doi: 10.1371/journal.ppat.1003747 (PMC3828167; doi:10.1371/journal.ppat.1003747)

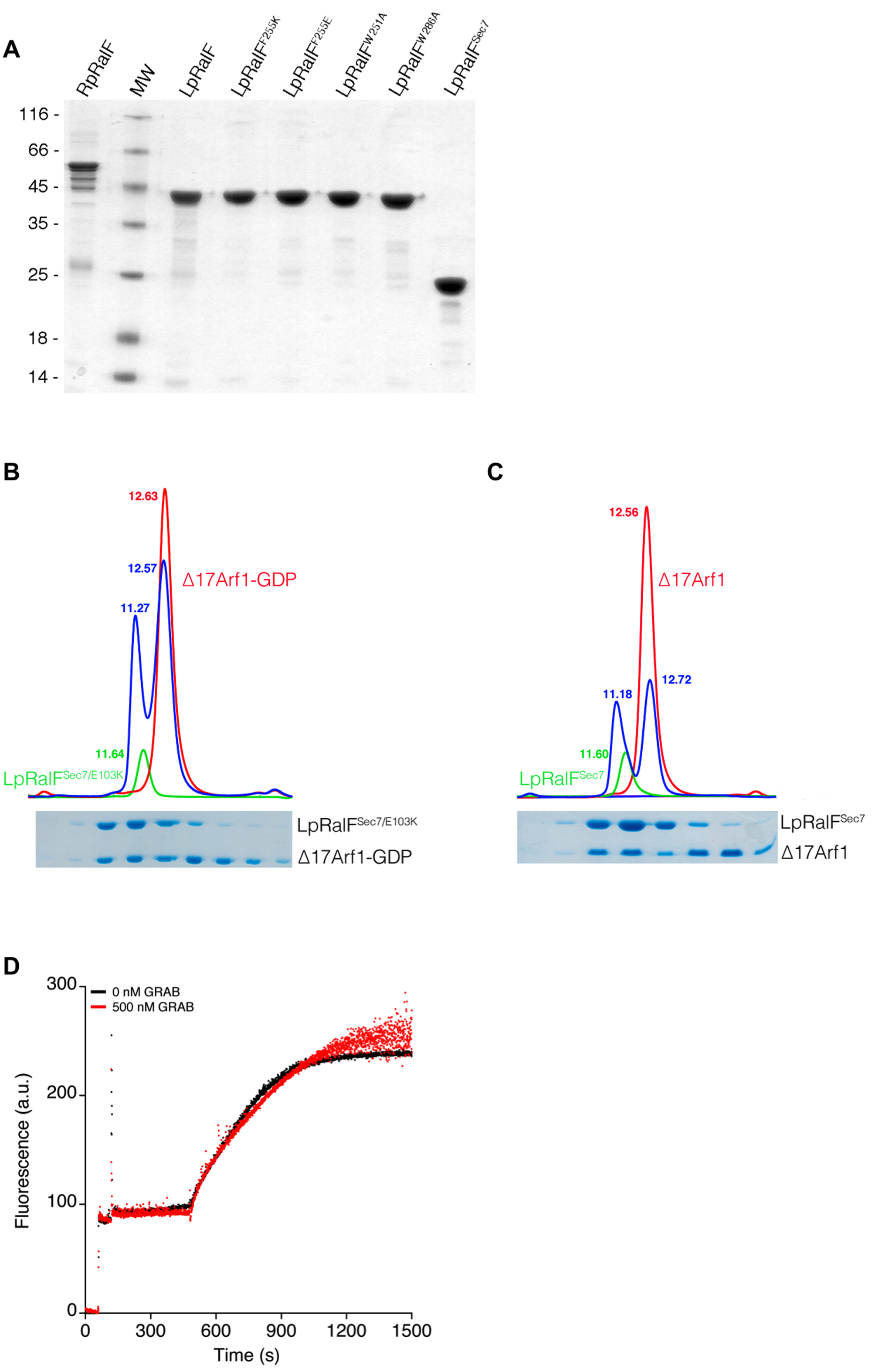

Supplement: Figure S1 — Characterization of constructs used in this study. A. SDS-PAGE analysis of LpRalF and RpRalF constructs used in this study. B. LpRalFSec7 E103K forms a stable complex with Arf1-GDP. The size exclusion chromatography profile of Arf1-GDP alone is in red, of LpRalF alone in green, and of Arf1 and LpRalF in blue. Below: SDS-PAGE analysis of the Arf1/LpRalFSec7 E103K experiment. C. LpRalFSec7 forms a stable complex with nucleotide-free Arf1. Color coding and analysis are as in Figure S1B. D. LpRalF is not regulated by a feed-back loop. Nucleotide exchange kinetics were analyzed in the presence of the Arf1-binding region of the Arf effector GRAB, which should deplete Arf1-GTP as it is produced by RalF. The nucleotide exchange experiment was carried out with liposomes (200 µM), myrArf1-GDP (0.4 µM), LpRalF (0.1 µM) with or without addition of GRAB. (TIF) [file ppat.1003747.s001.tif]

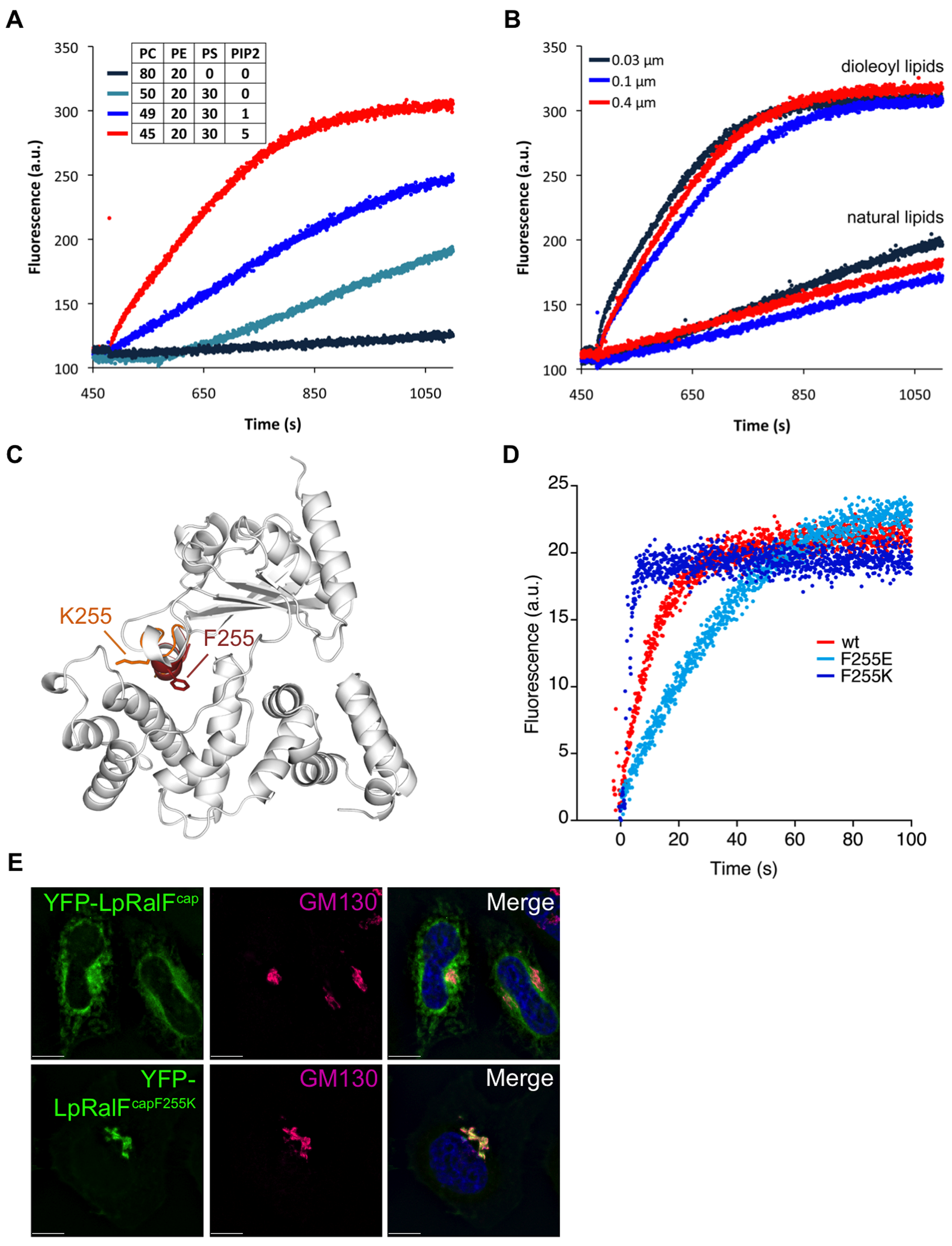

Supplement: Figure S2 — The aromatic cluster of Legionella pneumophila RalF is a membrane sensor. A–B. LpRalF is sensitive to liposome composition and packing defects but not to curvature. Representative fluorescence kinetics of Arf1 activation using liposomes of indicated compositions and curvatures. All experiments were carried out with liposomes (200 µM) extruded through a 0.1 µm filter unless indicated otherwise, myrArf1-GDP (0.4 µM), LpRalF (0.1 µM) and were started with 40 µM GTP. C. The crystal structure of LpRalFF255K retains the auto-inhibited conformation. The location of aromatic cluster is shown in magenta for LpRalFF255K, in red for LpRalF. D. Representative fluorescence kinetics of the F255 LpRalF mutants in the presence of liposomes. Experiments were done with 0.05 µM LpRalF, 0.4 µM myrArf1 and 200 µM liposomes (composition as in Figure 1D ). E. Localization of LpRalF and LpRalFF255K capping domains when ectopically expressed in HeLa cells. Cells transfected with YFP-LpRalFcap or YFP-LpRalFcapF255K were fixed 24 hours after transfection, stained with anti-GM130 antibodies (red) and DAPI (blue) and analyzed by fluorescence microscopy. Bar = 5 µm. (TIF) [file ppat.1003747.s002.tif]

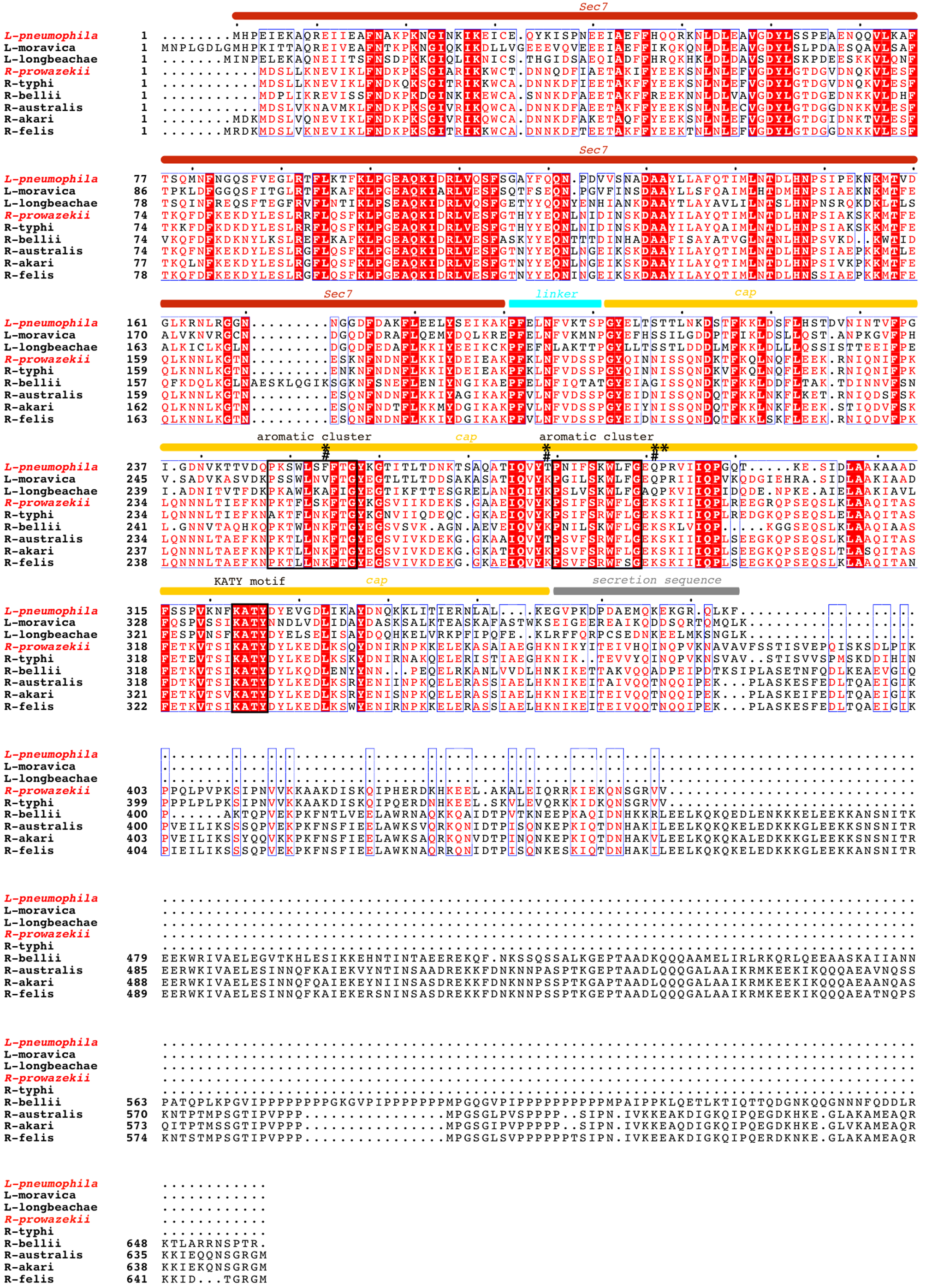

Supplement: Figure S3 — Alignment of RalF sequences from various Legionella and Rickettsia species. Residues in LpRalF permuted to the corresponding RpRalF residues are labelled with *. Residues in RpRalF permuted to the corresponding LpRalF residues are labelled with #. Done with MultAlin and drawn with ESPript. (TIF) [file ppat.1003747.s003.tif]

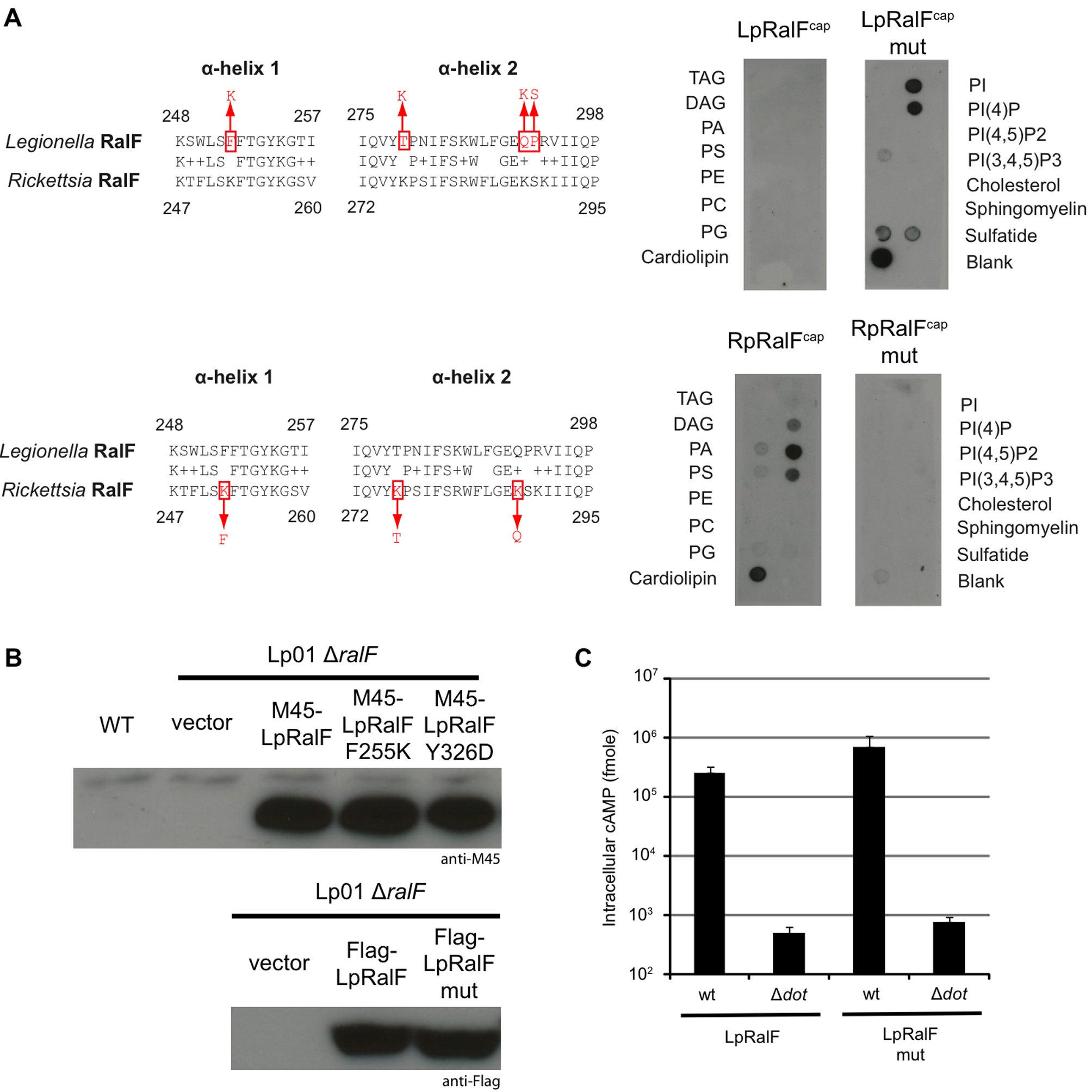

Supplement: Figure S4 — Characterization of LpRalF and RpRalF mutants. A. Alignment of LpRalF and RpRalF α-helices forming the aromatic cluster and lipid overlay assay. Divergent residues mutated in LpRalF (top) and RpRalF (bottom) in the subsequent experiments are shown with a red square. A protein-lipid overlay assay shows that mutations in the aromatic cluster modify lipid-binding properties of LpRalF (A) and RpRalF (B) capping domains. The binding of wild-type and mutants MBP-tagged capping domain to indicated lipids immobilized on nitrocellulose membranes was analyzed using an anti-MBP antibody. B. Expression of tagged proteins used to complement Lp01 ΔralF . Western blot on L. pneumophila Lp01 crude extracts expressing different constructs used in this study. C. Similar translocation of LpRalF and LpRalFmut by Legionella type IV secretion system. HEK293-FcγRII cells were infected with L. pneumophila wt or ΔdotA carrying a plasmid encoding the indicated Cya fusion proteins. cAMP level in the cell cytosol was quantified 1 h post-infection. Data are mean ± SD from three independent samples. (TIF) [file ppat.1003747.s004.tif]
