# Supplementary material for: A Novel Membrane Sensor Controls the Localization and ArfGEF Activity of Bacterial RalF
Source: PLoS Pathog. 2013 Nov 14;9(11):e1003747. doi: 10.1371/journal.ppat.1003747 (PMC3828167; doi:10.1371/journal.ppat.1003747)
Supplement: Table S1 — Data collection and refinement statistics of the LpRalFF255K mutant crystal structure. (DOCX) [file ppat.1003747.s005.docx]

**Table S1**

| Data collection statistics | | |
| --- | --- | --- |
| Space group | | P 3_1_ 2 1 |
| Cell parameters | | 78.26 78.26 115.01 90 90 120 |
| Wavelength (Å) | | 0.98 |
| Resolution range (Å) | | 39.13 – 3.1 (3.27 – 3.1) |
| Completness | | 99.8 (99.9) |
| I/σ(I) | | 24.2 (4.1) |
| Rmerge (%) | | 6.1 (62) |
| Refinement statistics | | |
| Resolution range (Å) | | 39.13 (3.1) |
| No of reflections | | 7785 |
| Rwork / Rfree (%) | | 19.77 / 28.05 |
| RMSD | Bond length (Å) | 0.010 |
|  | Bond Angle (°) | 1.25 |
| **Ramachandran** | Favoured (%) | 93.9 |
|  | Allowed (%) | 5.2 |
